# Diferenças Raciais no Controle da Pressão Arterial em Usuários de Anti-Hipertensivos em Monoterapia: Resultados do Estudo ELSA-Brasil

**DOI:** 10.36660/abc.20201180

**Published:** 2022-03-10

**Authors:** Camila Tavares Sousa, Antonio Ribeiro, Sandhi Maria Barreto, Luana Giatti, Luisa Brant, Paulo Lotufo, Dora Chor, Antônio Alberto Lopes, Sotero Serrate Mengue, André Oliveira Baldoni, Roberta Carvalho Figueiredo

**Affiliations:** 1 Universidade Federal de São João Del-Rei Divinópolis MG Brasil Universidade Federal de São João Del-Rei - Campus Centro-Oeste Dona Lindu, Divinópolis, MG – Brasil; 2 Universidade Federal de Minas Gerais Faculdade de Medicina Hospital das Clínicas Belo Horizonte MG Brasil Universidade Federal de Minas Gerais - Faculdade de Medicina e Hospital das Clínicas, Belo Horizonte, MG – Brasil; 3 Universidade Federal de Minas Gerais Belo Horizonte MG Brasil Universidade Federal de Minas Gerais - Medicina Preventiva e Social, Belo Horizonte, MG – Brasil; 4 Universidade de São Paulo Departamento de Medicina São Paulo SP Brasil Universidade de São Paulo - Departamento de Medicina, São Paulo, SP – Brasil; 5 Fundação Oswaldo Cruz Escola de Saúde Pública Rio de Janeiro RJ Brasil Fundação Oswaldo Cruz - Escola de Saúde Pública, Rio de Janeiro, RJ – Brasil; 6 Universidade Federal da Bahia Faculdade de Medicina Salvador BA Brasil Universidade Federal da Bahia - Faculdade de Medicina, Salvador, BA – Brasil; 7 Universidade Federal de Ciências da Saúde de Porto Alegre Programa de Pós-Graduação em Epidemiologia Porto Alegre RS Brasil Universidade Federal de Ciências da Saúde de Porto Alegre - Programa de Pós-Graduação em Epidemiologia, Porto Alegre, RS – Brasil

**Keywords:** Anti-Hipertensivos, Hipertensão, Grupos de Populações Continentais

## Abstract

**Fundamento:**

Aparentemente, a pior resposta a algumas classes de anti-hipertensivos, especialmente inibidores da enzima conversora da angiotensina e bloqueadores de receptor de angiotensina, pela população negra, explicaria, pelo menos parcialmente, o pior controle da hipertensão entre esses indivíduos. Entretanto, a maioria das evidências vêm de estudos norte-americanos.

**Objetivos:**

Este estudo tem o objetivo de investigar a associação entre raça/cor da pele autorrelatadas e controle de PA em participantes do Estudo Longitudinal de Saúde do Adulto (ELSA-Brasil) utilizando várias classes de anti-hipertensivos em monoterapia.

**Métodos:**

O estudo envolveu uma análise transversal, realizada com participantes da linha de base do ELSA-Brasil. O controle de pressão arterial foi a variável de resposta, participantes com valores de PA ≥140/90 mmHg foram considerados descontrolados em relação aos níveis de pressão arterial. A raça/cor da pele foi autorrelatada (branco, pardo, negro). Todos os participantes tiveram que responder perguntas sobre uso contínuo de medicamentos. A associação entre o controle de PA e raça/cor da pele foi estimada por regressão logística. O nível de significância adotado nesse estudo foi de 5%.

**Resultados:**

Do total de 1.795 usuários de anti-hipertensivos em monoterapia na linha de base, 55,5% se declararam brancos, 27,9%, pardos e 16,7%, negros. Mesmo depois de padronizar em relação a variáveis de confusão, negros em uso de inibidores da enzima conversora de angiotensina (IECA), bloqueadores de receptor de angiotensina (BRA), diuréticos tiazídicos (DIU tiazídicos) e betabloqueadores (BB) in monoterapia tinham controle de pressão arterial pior em comparação a brancos.

**Conclusões:**

Os resultados deste estudo sugerem que, nesta amostra de brasileiros adultos utilizando anti-hipertensivos em monoterapia, as diferenças de controle de pressão arterial entre os vários grupos raciais não são explicadas pela possível eficácia mais baixa dos IECA e BRA em indivíduos negros.

## Introdução

Vários estudos demonstraram a prevalência e a gravidade da hipertensão são mais altas em negros que em brancos.^1^ Além disso, os dados indicam que, entre os pacientes hipertensos, os negros têm, em geral, um controle pior da pressão arterial que os brancos.^1^ A diferença no controle da pressão arterial entre brancos e negros parece ser maior para algumas classes de anti-hipertensivos.^2^ A hipertensão afeta desproporcionalmente mais indivíduos negros. Além disso, o controle dos níveis de pressão arterial também parece ser mais difícil nesses indivíduos em comparação com a população branca.^1^ Aparentemente, a pior resposta a algumas classes de anti-hipertensivos explicaria, pelo menos parcialmente, o pior controle da hipertensão entre esses indivíduos.^2^

Estudos mostram que um parte da população negra tem baixa produção de renina e, portanto, por um mecanismo compensatório, o corpo aumenta a produção vascular de angiotensina II, e, consequentemente, há uma aumento nos efeitos da aldosterona.^2 , 3^ Vários estudo de monoterapia indicam que pacientes negros têm menos redução da pressão arterial (PA) com inibidores de enzima conversora da angiotensina (IECA) ou bloqueadores de receptor de angiotensina (BRA) em comparação a pacientes brancos.^4^ Além disso, ao comparar as classes de bloqueadores dos canais de cálcio (BCC) e diuréticos tiazídicos (DIU tiazídicos) indicados como primeira escolha para o tratamento da hipertensão na população negra, o uso de IECA em monoterapia estava associado ao aumento do risco de eventos cardiovasculares nesses indivíduos.^8^

Nesse sentido, as diretrizes terapêuticas norte-americanas e europeias não recomendam IECA ou BRA como monoterapia, como medicamento de escolha no tratamento da hipertensão em indivíduos negros, já que são medicamentos que agem na via renina-angiotensina-aldosterona.^11 , 12^

Entretanto, embora o tratamento da PA tenha sido amplamente estudado em afro-americanos,^1 , 2 , 5 , 6^ isso não se aplica a negros brasileiros. Ainda há grande escassez de estudos sobre esse assunto no país, e, dessa forma, os dados são extrapolados especialmente dos Estados Unidos da América (EUA). Entretanto, essa extrapolação exige certo cuidado, já que há diferenças entre a população negra norte-americana e a brasileira, especialmente em relação à alta miscigenação,^13^ às condições socioeconômicas brasileiras, e ao risco cardiovascular,^14 , 15^ o que faz com que esse campo seja uma área de pesquisa importante.

Os BRA e IECA estão entre os anti-hipertensivos mais frequentemente usados entre os adultos brasileiros,^16^ independentemente de raça/cor da pele, principalmente porque são distribuídos gratuitamente pelo Sistema Único de Saúde brasileiro (SUS). Os resultados da Pesquisa Nacional sobre o Acesso, Utilização e Promoção do Uso Racional de Medicamentos no Brasil (PNAUM), demonstraram que cerca de 21% dos respondentes utilizavam IECA (enalapril ou captopril), e 20% usavam BRA (losartana).^17^ A prevalência do uso de monoterapia com IECA e BRA na linha de base do Estudo Longitudinal de Saúde do Adulto (ELSA-Brasil) foi 12,4% e 11,0%, respectivamente.^16^ Portanto, o presente estudo tem o objetivo de investigar a associação entre raça/cor da pele autorrelatadas e controle de PA em participantes do ELSA-Brasil utilizando várias classes de anti-hipertensivos em monoterapia.

## Métodos

### Desenho e população do estudo

O ELSA-Brasil é uma coorte prospectiva composta de 15.105 funcionários públicos, na ativa ou aposentados, de sete instituições públicas ou de educação superior e/ou pesquisa de sete capitais brasileiras. Mais informações sobre o desenho do estudo e o perfil da coorte podem ser encontradas nos artigos publicados por Aquino et al.^18^ e Schmidt et al.^19^

O estudo envolveu uma análise transversal, realizada com participantes da linha de base (2008-2010) do ELSA-Brasil. Todos os participantes que eram usuários de IECA, BRA, BCC, betabloqueadores (BB) e DIU tiazídicos em monoterapia, que responderam ao questionário sobre o uso de medicamentos, tinham informações disponíveis sobre raça/cor da pele autorrelatadas, e sobre os valores dos níveis de pressão arterial, foram incluídos.

Dos 4.412 participantes que usavam anti-hipertensivos, os participantes que não apresentaram informações sobre raça/cor da pele autorrelatadas (n = 56) e os que se declararam asiáticos ou indígenas (n = 154) foram excluídos, além dos participantes que usaram anti-hipertensivos em politerapia (n = 2.407). Portanto, a amostra analítica foi composta de 1.795 usuários de anti-hipertensivo em monoterapia. Todos os participantes assinaram um formulário de consentimento informado e o estudo foi aprovado pelos comitês de ética de cada instituição envolvida.

### Variáveis do estudo

#### Controle da pressão arterial

Os níveis de pressão arterial foram medidos após um repouso de cinco minutos, com os participantes sentados em uma sala silenciosa em temperatura controlada. O dispositivo com manguito duplo e oscilométrico (Omron HEM 705CPINT) foi utilizado.^20 , 21^ Foram feitas três aferições em intervalos de um minuto e a média das duas últimas foi considerada a PA de cada participante.^21^ Os participantes foram classificados em dois grupos dependendo se tinham PA controlada ou não. A PA sistólica <140 mmHg e a PA diastólica <90 mmHg foram consideradas controladas. Participantes com valores de PA ≥140/90 mmHg foram considerados descontrolados em relação aos níveis de pressão arterial.^22^

#### Raça/Cor da pele autorrelatadas

Todos os participantes tiveram que responder: *“O censo brasileiro (IBGE) usa os termos ‘negro’, ‘pardo’, ‘branco’, ‘asiático’, e ‘indígena’ para classificar a cor da pele ou a raça das pessoas. Se você tivesse que responder ao censo do IBGE hoje, como você se classificaria em relação a sua raça ou cor?”* , com as seguintes opções de resposta: negro, pardo, branco, asiático e indígena brasileiro. No presente estudo, apenas participantes que alegaram ser brancos, negros ou pardos foram incluídos, devido aos números baixos das outras categorias.

#### Classe de anti-hipertensivos

Todos os participantes tiveram que responder perguntas sobre uso contínuo de medicamentos nas duas semanas anteriores^23^ e foram instruídos a levar as prescrições e/ou os medicamentos usados, ao centro de pesquisa.

Os medicamentos anti-hipertensivos relatados pelos participantes foram classificados de acordo com as seguintes classes: bloqueadores de receptor de angiotensina (BRA) (candesartana, irbesartana, losartana, olmesartana, telmisartana, valsartana); betabloqueadores (betabloqueadores com seletividade beta-1 (atenolol, bisoprolol, nebivolol, metropolol) e bloqueadores não seletivos (propanolol, nadolol, pindolol)); bloqueadores dos canais de cálcio diidropiridínicos (amlodipina, felodipina, isradipina, lacidipina, lercanidipino, nifedipino, nimodipino, nitrendipino, manidipino) e não diidropiridínicos (diltiazem, verapamil); diuréticos tiazídicos (clortalidona, hidroclorotiazida, indapamida); e inibidores de enzima conversora da angiotensina (captopril, benazepril, delapril, fosinopril, lisinopril, enalapril, perindopril, ramipril, trandolapril).

#### Características demográficas e socioeconômicas, estilos de vida relacionados à saúde, condições antropométricas e clínicas

As informações sobre as características demográficas socioeconômicas dos participantes foram obtidas por questionários estruturados.^18^ No presente estudo, as seguintes variáveis sociodemográficas foram consideradas: sexo, idade (em escala contínua), e grau de instrução (categorizados em: Ensino superior completo, Ensino médio completo < Ensino médio completo)

O consumo excessivo de bebidas alcoólicas foi avaliado e definido utilizando tipo de bebida geralmente consumida, frequência, e padrões de consumo. As informações obtidas no questionário foram resumidas e definidas em gramas de álcool consumidos por semana. Consumo excessivo >210 g de álcool por semana foi considerado para homens, e >140 g por semana, para mulheres.^24^

O índice de massa corporal (IMC) (kg/m^2^) foi obtido medindo-se a altura e o peso e classificado em três categorias: <25 (peso normal); ≥25 e <30 (sobrepeso) e ≥30 (obesidade). Diabetes Mellitus (DM) foi definida por autorrelato de diagnóstico anterior ou uso de medicamento para tratar o diabetes; ou glicemia em jejum ≥126 mg/dL; ou teste de tolerância a glicose ≥200 mg/dL; ou hemoglobina glicada ≥6,5 %.^25^

Todos os participantes responderam há quanto tempo estavam usando o medicamento anti-hipertensivo relatado. O tempo foi classificado como anos de uso.

#### Análise estatística

Inicialmente, as características demográficas e socioeconômicas, hábitos de vida relacionados à saúde, condições antropométricas e clínicas dos participantes foram distribuídas de acordo com a população total e as três categorias de raça/cor da pele autorrelatadas. Elas foram descritas utilizando proporções para variáveis categóricas, e média e desvio padrão para variáveis contínuas. A comparação entre grupos foi realizada usando o teste t Student não pareado para variáveis categóricas e teste ANOVA de via única para variáveis contínuas. A associação entre o controle de PA e raça/cor da pele foi estimada por regressão logística.

As covariáveis (características demográficas e socioeconômicas, consumo excessivo de álcool, IMC (contínuo), DM, e tempo de uso de anti-hipertensivos) foram adicionadas aos modelos passo a passo com eliminação posterior. As razões de chance (RC) brutas e padronizadas e seus respectivos intervalos de confiança de 95% (IC 95%) foram estimadas. Foi investigado se a raça/cor da pele autorrelatada (categoria de referência: brancos) estava associada ao controle de PA entre os 1.795 usuários das cinco classes de anti-hipertensivos em monoterapia da linha de base. Após a análise univariada, as RC brutas (Modelo 0) foram padronizadas para idade, sexo, e grau de instrução (Modelo 1). Em seguida, o Modelo 1 foi padronizado para consumo excessivo de álcool (Modelo 2), e, por último, o Modelo 2 foi padronizado para IMC, DM, e tempo de uso de anti-hipertensivos (Modelo 3). Todas as variáveis que permaneceram estatisticamente associadas à variável de resposta (p <0,05) foram mantidas no modelo final após todas as padronizações. Todas as análises foram realizadas utilizando o software Stata (versão 14.0)

## Resultados

Do total de 1.795 usuários de anti-hipertensivos em monoterapia na linha de base, 995 (55,5%) se declararam brancos. Tanto no total da população quanto nos três grupos raciais, as mulheres eram maioria. A média de idade entre os brancos era de 57 (9,0) anos, e era de 55 (8,2) anos entre as populações negra e parda. O ensino superior completo era significativamente mais frequente entre os brancos em comparação às populações negra parda. A frequência de DM e obesidade era significativamente mais alta entre indivíduos negros, seguida dos participantes pardos e brancos. O consumo excessivo de álcool não era significativamente diferente entre os três grupos raciais ( Tabela 1 ).

**Tabela 1:** – Distribuição de usuários de anti-hipertensivos em monoterapia na linha de base de acordo com características socioeconômicas; hábitos relacionados à saúde e presença de morbidades; controle da pressão arterial; níveis de pressão arterial; classe de fármaco e tempo de uso dos anti-hipertensivos distribuídos de acordo com categorias de raça/cor da pele autorrelatadas. n (%). média (DP) ELSA-Brasil*. (2008-2010) (N= 1,795)*

Variáveis	Geral (N=1,795)	Brancos (N=995)	Pardos (N=501)	Negros (N=299)	p-valor^‡^
**Sexo**	832 (46,4)	486 (48,8)	230 (45,9)	116 (38,8)	0,009
Masculino	963 (53,6)	509 (51,2)	271 (54,1)	183 (61,2)	
Feminino					
**Idade (anos)**	56 (8,7)	57 (9,0)	55 (8,2)	55 (8,1)	0,023^§^
**Grau de instrução**					
Ensino superior completo	972 (54,1)	699 (70,2)	203 (40,5)	70 (23,4)	0,001
Ensino médio completo	583 (32,5)	228 (22,9)	205 (40,9)	150 (50,2)	
< Ensino médio completo	240 (13,4)	68 (6,8)	93 (18,6)	79 (26,4)	
**Consumo excessivo de álcool** ^**1**^					
Não	1,650 (92,0)	902 (90,7)	467 (93,4)	281 (94,0)	0,079
Sim	143 (8,0)	92 (9,3)	33 (6,6)	18 (6,0)	
**Diabetes**					
Não	1,273 (71,0)	735 (74,0)	348 (69,5)	190 (63,5)	0,002
Sim	521 (29,0)	259 (26,0)	153 (30,5)	109 (36,5)	
**Índice de massa corporal (IMC)**					
Peso normal	458 (25,5)	267 (26,8)	132 (23,3)	59 (19,8)	0,012
Sobrepeso	780 (43,5)	444 (44,6)	212 (42,3)	124 (41,6)	
Obesidade	556 (31,0)	284 (28,5)	157 (31,4)	115 (39,6)	
**Controle da pressão arterial**					
Controlada	1,297 (72,3)	776 (78,0)	338 (67,5)	183 (61,2)	0,001
Descontrolada	498 (27,7)	219 (22,0)	163 (32,5)	116 (38,8)	
**Média dos níveis de pressão arterial sistólica**	128 (17,3)	126 (16,7)	130 (16,5)	133 (19,2)	0,004
**Média dos níveis de pressão arterial diastólica**	79 (10,5)	81 (10,5)	81 (10,5)	82 (10,8)	0,362
**Classe de anti-hipertensivos**					
IECA	500 (27,9)	266 (26,7)	142 (28,3)	92 (30,8)	0,001
DIU tiazídicos	291 (16,2)	123 (12,4)	98 (19,6)	70 (23,4)	
BCC	121 (6,7)	51 (5,1)	37 (7,4)	33 (110,0)	
BRA	439 (24,5)	278 (28,0)	114 (22,7)	47 (15,7)	
BB	444 (24,7)	277 (27,8)	110 (22,0)	57 (19,1)	
Tempo de uso de anti-hipertensivos (anos)	4,0 (4,3)	4,2 (4,2)	3,9 (4,5)	3,5 (4,1)	0,106^§^

*Diferenças no N total para cada variável se devem aos valores faltantes. ^†^ Estudo Longitudinal de Saúde do Adulto (ELSA-Brasil). ^1^ Consumo excessivo de álcool definido como >210 g de álcool/semana para homens, e >140 g de álcool/semana para mulheres. ^2^ Valores de referência para pressão arterial de controle: Controlada (<140/90 mmHg), Descontrolada (≥140 / 90 mmHg) ^‡^ p-valor resultante do Teste qui-quadrado ^§^ p-valor resultante do teste ANOVA. Pressão arterial (PA), Inibidores da enzima conversora de angiotensina (IECA), Bloqueadores de receptor de angiotensina (BRA), Bloqueadores de canal de cálcio (BCC), Betabloqueadores (BB) Diuréticos tiazídicos (DIU tiazídicos).*

A porcentagem de participantes que tinham PA descontrolada era mais alta entre os indivíduos negros, seguida dos participantes pardos e brancos (38,8 %, 32,5 %, e 22,0 % respectivamente; p <0,05). Os participantes negros tinham uma frequência mais alta de uso de IECA (30,8 %), DIU tiazídicos (23,4 %) e BCC (11,0 %) quando comparados com outras raças. A porcentagem de uso de BRA (28,0 %) e BB (27,8 %) era mais alta entre os participantes brancos ( Tabela 1 ).

A porcentagem de participantes que não tinham controle de PA era mais alta entre os usuários de IECA, seguida dos usuários de BCC, DIU tiazídicos, BRA e BB (33,2 %, 31,4 %, 28,2 %, 26,9 %, e 21,2 % respectivamente; p <0,05). Níveis de pressão arterial sistólica mais altos estavam presentes entre os usuários de BCC, seguidos de IECA. Usuários de IECA tinham níveis de pressão arterial diastólica médios mais altos, seguidos dos usuários de DIU tiazídicos e BRA. O tempo médio de uso de anti-hipertensivos era mais alto entre os usuários de BCC ( Tabela 2 ). Mais informações sobre a distribuição dos participantes do estudo por raça/cor da pele autorrelatadas e classes de anti-hipertensivos são apresentadas na tabela 1 do apêndice.

**Tabela 2:** – Distribuição de usuários de anti-hipertensivo em monoterapia na linha de base de acordo com características socioeconômicas; hábitos relacionados à saúde e presença de morbidades; controle da pressão arterial; níveis de pressão arterial e tempo de uso dos anti-hipertensivos distribuídos de acordo com classes de anti-hipertensivos. n (%). média (DP) ELSA-Brasil†. (2008-2010) (N= 1,795)*

Variáveis	IECA (N=500)	Diuréticos tiazídicos (N=291)	BCC (N=121)	BRA (N=439)	BB (N=444)	p-valor^‡^
**Sexo**						
Masculino	289 (57,8)	84 (28,9)	59 (48,8)	227 (51,7)	173 (38,9)	0,001
Feminino	211 (42,2)	207 (71,1)	62 (51,2)	212 (48,3)	271 (61,1)	
Idade (anos)	55 (8,4)	55 (8,5)	55 (8,5)	57 (8,6)	55 (8,9)	0,668^§^
**Grau de instrução**						
Ensino superior completo	230 (46,0)	119 (40,9)	65 (53,7)	291 (53,7)	267 (60,1)	0,001
Ensino médio completo	188 (37,6)	114 (39,2)	36 (29,7)	112 (25,5)	133 (23,0)	
< Ensino médio completo	82 (16,4)	58 (19,9)	20 (16,5)	36 (8,2)	44 (9,9)	
**Consumo excessivo de álcool^1^**						
Não	449 (89,8)	275 (94,8)	113 (93,4)	400 (91,3)	413 (93,0)	0,104
Sim	51 (10,2)	15 (5,2)	8 (6,6)	38 (8,7)	31 (7,0)	
Diabetes						
Não	307 (61,4)	219 (75,3)	83 (68,6)	306 (69,7)	358 (80,8)	0,001
Sim	193 (38,6)	72 (24,7)	38 (31,4)	133 (30,3)	85 (19,2)	
**Índice de massa corporal (IMC)**						
Peso normal	115 (23,0)	75 (25,8)	38 (31,4)	93 (21,2)	137 (30,9)	0,001
Sobrepeso	212 (42,4)	102 (35,1)	55 (45,5)	206 (46,9)	205 (46,3)	
Obesidade	173 (34,6)	114 (39,2)	28 (23,1)	140 (31,9)	101 (22,8)	
**Controle da pressão arterial**						
Controlada	334 (66,8)	209 (71,8)	83 (68,6)	321 (73,1)	350 (78,8)	0,001
Descontrolada	166 (33,2)	82 (28,2)	38 (31,4)	118 (26,9)	94 (21,2)	
Média dos níveis de pressão arterial sistólica	130 (18,6)	128 (15,4)	131 (15,1)	128 (16,5)	125 (17,7)	0,001^§^
Média dos níveis de pressão arterial diastólica	82 (11,3)	80 (9,14)	79 (10,0)	80 (9,9)	77 (10,8)	0,001^§^
Tempo de uso de anti-hipertensivos (anos)	4,5 (4,5)	3,6 (4,5)	4,8 (4,5)	2,6 (2,6)	4,7 (4,9)	0,001

*Diferenças no N total para cada variável se devem aos valores faltantes. ^†^ Estudo Longitudinal de Saúde do Adulto (ELSA-Brasil). ^1^ Consumo excessivo de álcool definido como >210 g de álcool/semana para homens, e >140 g de álcool/semana para mulheres. ^2^ Valores de referência para pressão arterial de controle: Controlada (<140/90 mmHg), Descontrolada (≥140 / 90 mmHg) ^‡^ p-valor resultante do Teste qui-quadrado ^§^ p-valor resultante do teste ANOVA. Pressão arterial (PA), Inibidores da enzima conversora de angiotensina (IECA), Bloqueadores de receptor de angiotensina (BRA), Bloqueadores de canal de cálcio (BCC), Betabloqueadores (BB) Diuréticos tiazídicos (DIU tiazídicos)*

Ao investigar a associação entre a raça/cor da pele autorrelatada e o controle de PA entre os usuários de IECA em monoterapia, mesmo após a padronização para todas as variáveis, as chances de as populações parda e negra terem PA descontroladas eram 2,7 (IC 95%: 1,7;4,3) e 2,2 (IC 95%: 1,3;3,4) mais altas, respectivamente, quando comparadas com as da população branca. Entre os usuários de BRA, BB e DIU tiazídicos, apenas indivíduos negros tinham uma chance estatisticamente mais alta de ter PA descontrolada em comparação com os brancos, após a padronização para variáveis de confusão. Entre os usuários de BCC, a raça/cor da pele autorrelatada não estava estatisticamente associada à PA descontrolada ( Tabela 3 ).

**Tabela 3:** – Razões de chance (RC) brutas e padronizadas * no controle da pressão arterial+ de usuários de anti-hipertensivos em monoterapia na linha de base do ELSA-Brasil1 2008-2010 (n=1,795)

Classe de anti-hipertensivos	Multivariada			

	Modelo 0	Modelo 1	Modelo 2	Modelo 3
	RC (IC95%)	RC (IC95%)	RC (IC95%)	RC (IC95%)
**IECA (N=500)**				
Brancos	Ref.	Ref.	Ref.	Ref.
Pardos	2,9^**^(1,9;4,5)	2,8^**^(1,8;4,4)	2,8^**^(1,8;4,4)	2,7^**^(1,7;4,3)
Negros	2,5^**^(1,5;4,1)	2,3^**^(1,3;3,9)	2,3^**^(1,3;3,9)	2,2^**^(1,3;3,4)
**BRA (N=439)**				
Brancos	Ref.	Ref.	Ref.	Ref.
Pardos	1,3 (0,8;2.1)	1,1 (0,6;1.9)	1,1 (0,6;1.9)	1,2 (0,7;2.2)
Negros	2.4** (1,25;4.51)	1,9 (0,9;4.0)	2,0 (1,0;4.1)	2.2** (1,0;4.7)
BCC (N=121)				
**Brancos**	**Ref.**	**Ref.**	**Ref.**	**Ref.**
Pardos	0,8 (0,3;2.1)	0,7 (0,3;1.9)	0,7 (0,2;1.9)	0,7 (0,2;2.1)
Negros	1,3 (0,5;3.2)	1,0 (0,4;2.9)	1,0 (0,4;2.9)	1,1 (0,4;3.5)
**ΒB (N=444)**				
Brancos	Ref.	Ref.	Ref.	Ref.
Pardos	1,3 (0,8;2.3)	1,3 (0,7;2.3)	1,3 (0,7;2.3)	1,2 (0,6;2.2)
Negros	2,3^**^(1,2;4,3)	2,1^**^(1,0;4,1)	2,1^**^(1,0;4,2)	2.1^**^(1,0:4,4)
**DIU tiazídicos (N=291)**				
Brancos	Ref.	Ref.	Ref.	Ref.
Pardos	1,6 (0,9;3.0)	1,5 (0,8;2.9)	1,6 (0,8;3.2)	1,7 (0,9;3.4)
Negros	2.2^**^(1,2:4,2)	1,9 (1,0;4.0)	2,1^**^(1,0;4,5)	2,4^**^(1,1;5,1)

* Razões de chance (RC). ^+^ A categoria de referência é a pressão arterial controlada (<140/90 mmHg)** p <0,05. ^1^ Estudo Longitudinal de Saúde do Adulto (ELSA-Brasil). Modelo 1: Padronizado para idade, sexo e grau de instrução. Modelo 2: Em seguida, o Modelo 1 foi padronizado para consumo excessivo de álcool. Modelo 3: O Modelo 2 foi padronizado para IMC, diabetes mellitus, e tempo de uso de anti-hipertensivos. Inibidores da enzima conversora de angiotensina (IECA), Bloqueadores de receptor de angiotensina (BRA), Bloqueadores de canal de cálcio (BCC), Betabloqueadores (BB) Diuréticos tiazídicos (DIU tiazídicos)

## Discussão

Este estudo inova ao investigar disparidades raciais no controle da pressão arterial em usuários de classes diferentes de anti-hipertensivos em monoterapia em uma amostra com grande diversidade racial entre funcionários públicos brasileiros adultos. Nossos resultados não corroboram a maioria dos encontrados pelos estudos elaborados principalmente na população norte-americana,^8 , 12 , 13 , 26^ o que demonstra que usuários de IECA e BRA negros têm pior controle de pressão arterial em comparação com usuários de BB, BCC, e DIU tiazídicos. Os usuários negros de anti-hipertensivos em monoterapia da linha de base do ELSA-Brasil tinham uma chance maior de ter PA descontrolada não apenas nas classes IECA e BRA, mas em todas as outras, com exceção da classe BCC.

A diretriz norte-americana mais recente para tratamento de hipertensão^12^ recomenda incluir diuréticos tiazídicos ou bloqueadores dos canais de cálcio para adultos negros com hipertensão sem insuficiência cardíaca ou doença renal crônica. Essa recomendação é corroborada pelos resultados de estudos realizados com a população norte-americana, que demonstraram frequentemente que indivíduos negros, possivelmente por terem baixa produção de renina, têm pior controle da pressão arterial quando tratados com medicamentos que agem no sistema renina-angiotensina. Ademais, essa população tem resultados cardiovasculares piores quando tratada com esses anti-hipertensivos.^3 , 4 , 27 , 28^

Além da baixa produção de renina entre indivíduos negros, a resposta mais baixa dos IECA comparada com os DIU tiazídicos, BCC, e BB pode ser explicada por outros fatores. Já se sugeriu que essa baixa resposta é atribuída ao consumo alto de sódio por indivíduos negros mais sensíveis ao sal, em que a resposta a IECA seria enfraquecida de alguma maneira. Outros já sugeriram que a hipertensão na população negra pode não ter um mecanismo independente de angiotensina.^29^ Além disso, essa sensibilidade mais alta ao sal também pode explicar o melhor controle da pressão arterial entre os usuários negros de DIU tiazídicos.^30^ Outros estudos demonstraram um aumento significativo no risco de efeitos adversos associados a IECA em indivíduos negros, por exemplo a tosse, que contribui para um índice mais alto de interrupção do tratamento com IECA nesse grupo em comparação com outras raças.^31^

Nos anos 90, um estudo feito por Saunders e colaboradores demonstrou que, entre a população negra, a classe BCC, em comparação às classes BB e IECA, foi mais eficiente no controle dos níveis de pressão arterial diastólica e sistólica.^32^ Além disso, vários outros estudos mais recentes desenvolvidos principalmente entre negros norte-americanos, demonstraram que BCC, quando comparados a IECA, BRA e BB, foram mais eficientes na redução do risco de vários eventos cardiovasculares, tais como infarto agudo do miocárdio, acidente vascular cerebral, e insuficiência cardíaca.^8 , 11 , 12 , 26^ Neste estudo, entre os usuários de BCC em monoterapia, os indivíduos pardos e negros não tinham probabilidade maior de ter níveis de pressão arterial descontrolados em comparação aos brancos. Embora a ausência de associação possa ser explicada pelo baixo poder amostral nesse grupo (há apenas 121 usuários de BCC em monoterapia), os resultados estão alinhados à literatura que recomenda que os BCC sejam uma das primeiras escolhas de tratamento de hipertensão na população negra.

Nesse sentido, nossos resultados, que, em resumo, demonstraram que a maior chance de ter PA descontrolada em indivíduos negros não está restrita a usuários de IECA e BRA em monoterapia, sendo que também é encontrada entre usuários de DIU tiazídicos e BB, corroboram outros estudos que demonstraram que as explicações possíveis para que indivíduos negros tenham um controle de PA pior vão além da questão fisiológica que envolveria as classes de medicamento. Diferenças socioeconômicas, tais como grau de instrução mais baixo, são uma das principais determinantes da ocorrência do pior controle de hipertensão arterial,^33 , 34^ e podem explicar parcialmente as diferenças entre populações negras, pardas e brancas. Além disso, os contextos sociais ou “bairros” onde as pessoas moram podem contribuir significativamente para as disparidades raciais na saúde^35 , 36^ e podem ter um papel importante na explicação da relação entre raça/cor da pele e controle da hipertensão arterial.

Na verdade, estudos anteriores desenvolvidos na linha de base do ELSA-Brasil já demonstraram disparidades raciais na prevalência e no controle da hipertensão. Chor et al. demonstraram que indivíduos que declararam ser negros tinham pior controle da pressão arterial em comparação aos que se declaravam brancos, mesmo entre os usuários de anti-hipertensivos.^16^ Barber et al. investigaram a associação entre segregação residencial e fatores de risco cardiometabólico, que incluíam a presença da hipertensão. Os autores concluíram que, apesar de não existir diferença estatisticamente significativa, as populações negra e parda tinham mais probabilidade de viver em bairros economicamente segregados em relação aos brancos, e os indivíduos que moravam nesses bairros tinham uma probabilidade 26% maior de ter hipertensão.^36^ Além disso, Baldo e outros também mostram que participantes negros e pardos na linha de base do ELSA-Brasil tinham rigidez arterial maior se comparados aos brancos. Entretanto, essa diferença foi explicada pelos níveis médios de pressão arterial e pela idade dos participantes, sugerindo que as abordagens terapêuticas devem se concentrar no controle dos níveis de pressão arterial, especialmente entre os indivíduos negros.^37^

É importante destacar que, em nosso estudo, os participantes negros têm a frequência mais alta de uso de IECA, o que não seria esperado, já que tendemos a seguir as diretrizes baseadas nos estudos realizados com afro-americanos. Entretanto, as diretrizes também recomendam IECA ou BRA para indivíduos com diabetes,^11 , 12 , 22^ o que pode explicar esse resultado, já que os participantes negros neste estudo têm a frequência mais alta de DM.

Pena e outros demonstraram que, no Brasil, a cor da pele avaliada fenotipicamente tem uma correlação muito fraca com o grau de ancestralidade.^38^ Nesse sentido, resultados de ancestralidade ajudariam a entender melhor as disparidades raciais no controle da pressão arterial do ponto de vista genético. Entretanto, raça/cor da pele autorrelatada é um fenótipo que ultrapassa a genética e a experiência de vida, refletindo, portanto, as percepções do sujeito de seu pertencimento racial étnico.^39^

O presente trabalho inova ao investigar as disparidades raciais no controle da pressão arterial entre usuários de várias classes de anti-hipertensivos em uma amostra de funcionários públicos adultos brasileiros; entretanto, ele tem algumas limitações que devem ser destacadas. Primeiro, não havia informações sobre a dose do tratamento anti-hipertensivo, e sabe-se que existem diferenças na otimização da dose entre as várias classes de fármaco. Segundo, embora a monoterapia seja frequentemente mais usada para casos mais leves, o escalonamento da hipertensão arterial pode influenciar as opções terapêuticas, com algumas classes sendo mais indicadas no início do tratamento e outras, preferencialmente, em estágios mais avançados.^22^ Entretanto, não houve informações sobre o escalonamento da hipertensão. Terceiro, embora a PA descontrolada seja definida com base nos valores adotados pelas diretrizes nacionais e internacionais para o tratamento e controle da hipertensão, ela se baseou em uma medição específica dos níveis de pressão arterial. Nesse sentido, resultados falso-positivo e falso-negativo podem aparecer, o que poderia interferir em nossos resultados.

Quarto, embora nossos resultados sejam verdadeiros para a monoterapia, estudos demonstram que a baixa eficácia de IECA entre indivíduos negros é revertida pela associação desses medicamentos com DIU tiazídicos e BCC.^40 , 41^ Entretanto, devido ao baixo poder amostral, especialmente entre novos usuários, a terapia combinada não foi testada no presente estudo. Por último, embora tenham sido feitas padronizações para as principais variáveis, isso não controla as variáveis de confusão não medidas.

## Conclusão

Até onde sabemos, este é o primeiro estudo a investigar disparidades raciais entre os usuários de várias classes de anti-hipertensivos em monoterapia, em uma amostra de adultos brasileiros. Concluindo, os resultados deste estudo sugerem que as diferenças de controle de pressão arterial entre os vários grupos raciais não são explicadas pela possível eficácia mais baixa dos IECA e BRA em indivíduos negros, já que ela ocorre em outras classes de anti-hipertensivos. Esses resultados sugerem cautela nas tomadas de decisão do tratamento anti-hipertensivo com base apenas na raça dos pacientes, e apresentam informações relevantes que podem orientar a tomada de decisão para o tratamento e o controle da hipertensão arterial no contexto brasileiro, sugerindo que uma falta de controle de PA mais alta nos indivíduos negros pode estar mais relacionada a determinantes sociais que à classe de anti-hipertensivos utilizada. Políticas que atuem no acesso adequado ao tratamento e na educação dos pacientes, portanto, devem ser abordadas
