# Supplementary material for: Diferenças Raciais no Controle da Pressão Arterial em Usuários de Anti-Hipertensivos em Monoterapia: Resultados do Estudo ELSA-Brasil
Source: Arq Bras Cardiol. 2022 Mar 10;118(3):614–22. [Article in Portuguese] doi: 10.36660/abc.20201180 (PMC8959024; doi:10.36660/abc.20201180)
Supplement: Translation

# Translation: Racial Differences in Blood Pressure Control from Users of Antihypertensive Monotherapy: Results from the ELSA-Brasil Study

**Authors:** Camila Tavares Sousa, Antonio Ribeiro, Sandhi Maria Barreto, Luana Giatti, Luisa Brant, Paulo Lotufo, Dora Chor, Antônio Alberto Lopes, Sotero Serrate Mengue, André Oliveira Baldoni, Roberta Carvalho Figueiredo

**Affiliations:** 1 Brazil Universidade Federal de São João Del-Rei - Campus Centro-Oeste Dona Lindu, Divinópolis, MG – Brazil; 2 Brazil Universidade Federal de Minas Gerais - Faculdade de Medicina e Hospital das Clínicas, Belo Horizonte, MG – Brazil; 3 Brazil Universidade Federal de Minas Gerais - Medicina Preventiva e Social, Belo Horizonte, MG – Brazil; 4 Brazil Universidade de São Paulo - Departamento de Medicina, São Paulo, SP – Brazil; 5 Brazil Fundação Oswaldo Cruz - Escola de Saúde Pública, Rio de Janeiro, RJ – Brazil; 6 Brazil Universidade Federal da Bahia - Faculdade de Medicina, Salvador, BA – Brazil; 7 Brazil Universidade Federal de Ciências da Saúde de Porto Alegre - Programa de Pós-Graduação em Epidemiologia, Porto Alegre, RS – Brazil

**Keywords:** Antihypertensive Agents, Hypertension, Continental Population Groups

## Abstract

**Background:**

It seems that the worst response to some classes of antihypertensive drugs, especially angiotensin-converting enzyme inhibitors and angiotensin receptor blockers, on the part of the Black population, would at least partially explain the worse control of hypertension among these individuals. However, most of the evidence comes from American studies.

**Objectives:**

This study aims to investigate the association between self-reported race/skin color and BP control in participants of the Longitudinal Study of Adult Health (ELSA-Brasil), using different classes of antihypertensive drugs in monotherapy.

**Methods:**

The study involved a cross-sectional analysis, carried out with participants from the baseline of ELSA-Brasil. Blood pressure control was the response variable, participants with BP values ≥140/90 mmHg were considered out of control in relation to blood pressure levels. Race/skin color was self-reported (White, Brown, Black). All participants were asked about the continuous use of medication. Association between BP control and race/skin color was estimated through logistic regression. The level of significance adopted in this study was of 5%.

**Results:**

Of the total of 1,795 users of antihypertensive drugs in monotherapy at baseline, 55.5% declared themselves White, 27.9% Brown, and 16.7% Black. Even after adjusting for confounding variables, Blacks using angiotensin converting enzyme inhibitors (ACEI), angiotensin receptor blocker (ARB), thiazide diuretics (thiazide DIU), and beta-blockers (BB) in monotherapy had worse blood pressure control compared to Whites.

**Conclusions:**

Our results suggest that in this sample of Brazilian adults using antihypertensive drugs in monotherapy, the differences in blood pressure control between different racial groups are not explained by the possible lower effectiveness of ACEIs and ARBs in Black individuals.

## Introduction

Several studies have shown that the prevalence and severity of hypertension are higher in Blacks than in Whites;^1^ additionally, the data indicate that among hypertensive patients, Blacks, in general, have poorer blood pressure control than Whites.^1^ The Black-White difference in blood pressure control seems to be larger for some classes of antihypertensive drugs.^2^ Hypertension disproportionately affects more Black individuals; additionally, the control of blood pressure levels also seems more difficult in these individuals when compared to the White population^1^ . It seems that the worst response to some classes of antihypertensive drugs by the Black population would at least partially explain the worse control of hypertension among these individuals.^2^

Studies show that a portion of the Black population has a low production of renin; thus, by a compensatory mechanism, the body increases the vascular production of angiotensin II, and as a consequence there is an increase in the effects of aldosterone.^2 , 3^ Several monotherapy studies indicate that Black patients have less reduction in blood pressure (BP) with angiotensin-converting enzyme inhibitors (ACEIs) or angiotensin receptor blocker inhibitors (ARBs) compared to White patients.^4 - 7^ In addition, when comparing the classes of calcium channel blockers (CCBs) and thiazide diuretics (DIUs), indicated as the first choice for hypertension treatment in the Black population, the use of ACEIs in monotherapy was associated with an increased risk of cardiovascular events in these individuals.^8 - 10^

In this sense, the American, European therapeutic guidelines do not recommend ACEIs or ARBs as monotherapy, as first choice medication in the treatment of hypertension in Black individuals, since they are medications that act in the renin-angiotensin-aldosterone pathway.^11 , 12^

However, although the treatment of BP has been widely studied in African-Americans,^1 , 2 , 5 , 6^ the same is not true for Black Brazilians. There is still a great scarcity of studies on this topic in the country, and as such, we extrapolate the data mainly from the United States of America (USA). However, this extrapolation requires some caution, as there are differences between the American and the Brazilian Black populations, especially with regard to the high miscegenation in Brazil,^13^ socioeconomic conditions, and cardiovascular risk,^14 , 15^ which makes this field an important research area.

ARBs and ACEIs are among the most frequently used antihypertensive drugs among Brazilian adults,^16^ regardless of race/skin color, mainly because they are distributed free of charge by the Brazilian public health system (SUS). Results from the National Survey on Access, Use and Promotion of Rational Use of Medicines in Brazil (PNAUM), showed that about 21% of the respondents used ACEIs (enalapril or captopril) and 20% used ARB (losartana).^17^ The prevalence of ACEI and ARB monotherapy use in the Brazilian Longitudinal Study of Adult Health (ELSA-Brasil) baseline was 12.4 % and 11.0%, respectively.^16^ Thus, the present study aimed to investigate the association between self- reported race/skin color and BP control in participants of the ELSA-Brasil, using different classes of antihypertensive drugs in monotherapy.

## Methods

### Study design and Population

ELSA-Brasil is a prospective cohort composed of 15,105 public employees, active or retired, from seven public institutions of higher education and/or research from six Brazilian state capitals. More information on the study design and cohort profile can be found in the articles published by Aquino et al.^19^ and Schmidt et al.^20^

The present study involved a cross-sectional analysis, carried out with participants from the baseline (2008-2010) of ELSA-Brasil. All participants who were users of ACEIs, ARBs, CCBs, beta-blockers (BBs), and thiazide DIUs in monotherapy, who answered the questionnaire on the use of medications, had information available on self-reported race/skin color, and on the values of blood pressure levels, were included.

Of the 4,412 participants using antihypertensive drugs, participants who did not present information on self-reported race/skin color (n = 56) and those who declared themselves Asian or indigenous (n = 154) were excluded, in addition to those participants who used antihypertensive drugs in polytherapy (n = 2,407). Thus, the analytical sample was composed of 1,795 antihypertensive users in monotherapy. All participants signed an informed consent form, and the study was approved by the ethics committees of each institution involved.

### Study Variables

#### Blood pressure control

Blood pressure levels were measured after a five-minute rest, with the participant sitting in a quiet room at a controlled temperature. The two-way cuff and oscillometric device (Omron HEM 705CPINT) were used.^20 , 21^ Three measurements were taken after one-minute intervals, and the average of the last two was considered to be the BP of each participant.^21^ The participants were classified into two groups according to whether or not they had BP control. Those with systolic BP<140 mmHg and diastolic BP<90 mmHg were considered controlled. Participants with BP values≥140/90 mmHg were considered out of control in relation to blood pressure levels.^22^

#### Self-reported race/skin color

All participants were asked: “ *The Brazilian Census (IBGE) uses the terms ‘Black’, ‘Brown, ‘White’, ‘Asian’, and ‘indigenous’ to classify people’s skin color or race. If you had to answer the IBGE Census today, how would you rate yourself regarding your color or race?* ”, with the following response options: Black, Brown, White, Asian, and Brazilian Indigenous. In the present study, only participants who claimed to be White, Black, or Brown were included, due to low numbers of the other categories.

#### Class of antihypertensive drugs

All participants were asked about the continuous use of medication in the previous two weeks^23^ and were instructed to take prescriptions and/or medications used to the research center.

The antihypertensive medication reported by the participants were classified according to the following classes: angiotensin receptor blockers (ARBs) (candesartan, irbesartan, losartan, olmesartan, telmisartan, valsartan); Beta-blockers (beta blockers with beta-1 selectivity (atenolol, bisoprolol, nebivolol, metoprolol) and non-selectable blockers (propranolol, nadolol, pindolol)); dihydropyridine calcium channel blockers (amlodipine, felodipine, isradipine, lacidipine, lercanidipine, nifedipine, nimodipine, nitrendipino, manidipino) and non-dihydropyridine (diltiazem, verapamil); thiazide diuretics (chlortalidone, hydrochlorothiazide, indapamide); and angiotensin-converting enzyme inhibitors (captopril, benazepril, delapril, fosinopril, lisinopril, enalapril, perindopril, ramipril, trandolapril).

#### Demographic and socioeconomic characteristics, health-related lifestyles, anthropometric, and clinical conditions

Information on the demographic and socioeconomic characteristics of the participants was obtained through structured questionnaires.^18^ In the present study, the following sociodemographic variables were considered: sex, age (on a continuous scale), and education (categorized into: Undergraduate complete, Secondary complete, and < Secondary complete)

Excessive consumption of alcoholic beverages was assessed and defined using the type of drink usually consumed, frequency, and consumption patterns. The information obtained in the questionnaire was summarized and defined in grams of alcohol consumed per week. Excessive consumption >210 g of alcohol per week was considered for men, and >140 g per week for women.^24^

Body mass index (BMI) (kg/m^2^ ) was obtained by measuring height and weight, and was classified into three categories: <25 (normal weight); ≥25 and <30 (overweight); and ≥30 (obesity). Diabetes Mellitus (DM) was defined by self-report of previous diagnosis or use of medication to treat diabetes; by fasting glucose ≥126 mg/dL; by the glucose tolerance test ≥200 mg/dL; or by glycated hemoglobin ≥6.5 %.^25^

All participants answered how long they had been using the reported antihypertensive medication. Time was classified into years of use.

#### Statistical Analysis

Initially, the demographic and socioeconomic characteristics, health-related lifestyle habits, anthropometric, and clinical conditions of the participants were distributed according to the total population and the three self-reported race/skin color categories. They were described using proportions for categorical variables, and mean and standard deviation for continuous variables. The comparison between groups was performed using the chi-square test for categorical variables and the One-Way ANOVA test for continuous variables. Association between BP control and race/skin color was estimated through logistic regression.

The covariables (demographic and socioeconomic characteristics, excessive alcohol consumption, BMI (continuous), DM, and time of use of antihypertensive drugs) were entered into the models step by step with forward elimination. Crude and adjusted odds ratios (OR) and their respective 95% confidence intervals (95% CI) were estimated. We investigated whether self-reported race/skin color (reference category: Whites) was associated with BP control among the 1,795 users of the five classes of antihypertensive drugs in baseline monotherapy. After univariate analysis, the crude ORs (Model 0) were adjusted for age, sex, and education (Model 1). Model 1 was then adjusted for excessive alcohol consumption (Model 2), and finally Model 2 was adjusted for BMI, DM, and time of use of antihypertensive drugs (Model 3). All variables that remained statistically associated with the response variable (p <0.05) were maintained in the final model after all adjustments. All analyses were performed using software Stata (version 14.0).

## Results

Of the total of 1,795 users of antihypertensive drugs in monotherapy at baseline, 995 (55.5 %) declared themselves White. Both in the total population and in the three racial groups, women were the majority. The average age among Whites was 57 (9.0) years, and 55 (8.2) years between the Brown and Black populations. Complete higher education was significantly more frequent among the White as compared to the Black and Brown populations. The frequency of DM and obesity was significantly higher among Black individuals, followed by the Brown and White participants. Excessive alcohol consumption was not significantly different between the three racial groups ( Table 1 ).

**Table 1:** – Distribution of users antihypertensive in monotherapy at baseline according to socioeconomic characteristics; health-related lifestyle habits and presence of morbidities; control of blood pressure; blood pressure levels. class of drugs and time of use of antihypertensive drugs distributed according self-reported race/skin color categories. n (%). mean (SD) ELSA-Brasil*. (2008-2010) (N= 1.795)*

Variables	Overall (N=1.795)	White(N=995)	Brown (N=501)	Black (N=299)	p value^‡^
**Gender**	832 (46.4)	486 (48.8)	230 (45.9)	116 (38.8)	0.009
Male	963 (53.6)	509 (51.2)	271 (54.1)	183 (61.2)	
Female					
**Age (years)**	**56 (8.7)**	**57 (9.0)**	**55 (8.2)**	**55 (8.1)**	**0.023** ^**§**^
**Education**					
Undergraduate complete	972 (54.1)	699 (70.2)	203 (40.5)	70 (23.4)	0.001
Secondary complete	583 (32.5)	228 (22.9)	205 (40.9)	150 (50.2)	
< Secondary complete	240 (13.4)	68 (6.8)	93 (18.6)	79 (26.4)	
**Excessive drinking** ^**1**^					
No	1.650 (92.0)	902 (90.7)	467 (93.4)	281 (94.0)	0.079
Yes	143 (8.0)	92 (9.3)	33 (6.6)	18 (6.0)	
**Diabetes**					
No	1.273 (71.0)	735 (74.0)	348 (69.5)	190 (63.5)	0.002
Yes	521 (29.0)	259 (26.0)	153 (30.5)	109 (36.5)	
**Body mass index (BMI)**					
Normal weight	458 (25.5)	267 (26.8)	132 (23.3)	59 (19.8)	0.012
Overweight	780 (43.5)	444 (44.6)	212 (42.3)	124 (41.6)	
Obesity	556 (31.0)	284 (28.5)	157 (31.4)	115 (39.6)	
**Blood pressure control**					
Controlled	1.297 (72.3)	776 (78.0)	338 (67.5)	183 (61.2)	0.001
Out of control	498 (27.7)	219 (22.0)	163 (32.5)	116 (38.8)	
**Means of systolic blood pressure levels**	128 (17.3)	126 (16.7)	130 (16.5)	133 (19.2)	0.004
**Mean diastolic blood pressure levels**	79 (10.5)	81 (10.5)	81 (10.5)	82 (10.8)	0.362
**Class of antihypertensive drugs**					
ACEI	500 (27.9)	266 (26.7)	142 (28.3)	92 (30.8)	0.001
Thiazide DIU	291 (16.2)	123 (12.4)	98 (19.6)	70 (23.4)	
CCB	121 (6.7)	51 (5.1)	37 (7.4)	33 (110.0)	
ARB	439 (24.5)	278 (28.0)	114 (22.7)	47 (15.7)	
BB	444 (24.7)	277 (27.8)	110 (22.0)	57 (19.1)	
Time of use of antihypertensive drugs (years)	4.0 (4.3)	4.2 (4.2)	3.9 (4.5)	3.5 (4.1)	0.106^§^

*Differences in total N for each variable are due to missing values.^†^The Longitudinal Study of Adult Health (ELSA-Brasil).^1^Excessive drinking defined as >210 g alcohol/week for men and 140 g alcohol/week for women..^2^Reference values for blood pressure control: Controlled (<140/90 mmHg), Out of control (≥140 / 90 mmHg)^‡^p-value resulting from the Chi-square test^§^p-value resulting from the ANOVA test. Blood pressure (BP), Angiotensin converting enzyme inhibitors (ACEI), Angiotensin receptor blocker (ARB), Calcium channel blocker (BCC), Beta blocker (BB), Thiazide diuretic (Thiazide DIU)*

The percentage of participants who had uncontrolled BP was higher among Black individuals followed by the Brown and White participants (38.8 %, 32.5 %, and 22.0 % respectively; p <0.05). The Black participants had a higher frequency of use of ACEIs (30.8 %), thiazide DIUs (23.4 %), and CCBs (11.0 %), when compared to the other races. The percentage of use of ARBs (28.0 %) and BBs (27.8 %) was higher among the White participants ( Table 1 ).

The percentage of participants who had no BP control was higher among users of ACEIs, followed by users of CCBs, thiazide DIUs, ARBs, and BBs (33.2 %, 31.4 %, 28.2 %, 26.9 %, and 21.2% respectively; p <0.05). Higher systolic blood pressure levels were presented among CCB users, followed by ACEIs. Users of ACEIs had higher mean diastolic blood pressure levels, followed by users of thiazide DIUs and ARBs. The average time of use of antihypertensive drugs was higher among CCB users ( Table 2 ). More information on the distribution of study participants according to self-reported race/skin color and antihypertensive drugs classes can be seen in Table 1 of the appendix.

**Table 2:** – Distribution of users of antihypertensive drugs in monotherapy at baseline according to socioeconomic characteristics; health-related lifestyle habits and presence of morbidities; control of blood pressure; blood pressure levels and time of use of antihypertensive distributed according to how antihypertensive classes. n (%). mean (SD)ELSA-Brasil†. (2008-2010) (N= 1.795)*

Variables	ACEI (N=500)	Thiazide diuretic (N=291)	BCC (N=121)	ARB (N=439)	BB (N=444)	p value^‡^
**Gender**						
Male	289 (57.8)	84 (28.9)	59 (48.8)	227 (51.7)	173 (38.9)	0.001
Female	211 (42.2)	207 (71.1)	62 (51.2)	212 (48.3)	271 (61.1)	
Age (years)	55 (8.4)	55 (8.5)	55 (8.5)	57 (8.6)	55 (8.9)	0.668^§^
**Education**						
Undergraduate complete	230 (46.0)	119 (40.9)	65 (53.7)	291 (53.7)	267 (60.1)	0.001
Secondary complete	188 (37.6)	114 (39.2)	36 (29.7)	112 (25.5)	133 (23.0)	
< Secondary complete	82 (16.4)	58 (19.9)	20 (16.5)	36 (8.2)	44 (9.9)	
**Excessive drinking^1^**						
No	449 (89.8)	275 (94.8)	113 (93.4)	400 (91.3)	413 (93.0)	0.104
Yes	51 (10.2)	15 (5.2)	8 (6.6)	38 (8.7)	31 (7.0)	
Diabetes						
No	307 (61.4)	219 (75.3)	83 (68.6)	306 (69.7)	358 (80.8)	0.001
Yes	193 (38.6)	72 (24.7)	38 (31.4)	133 (30.3)	85 (19.2)	
**Body mass index (BMI)**						
Normal weight	115 (23.0)	75 (25.8)	38 (31.4)	93 (21.2)	137 (30.9)	0.001
Overweight	212 (42.4)	102 (35.1)	55 (45.5)	206 (46.9)	205 (46.3)	
Obesity	173 (34.6)	114 (39.2)	28 (23.1)	140 (31.9)	101 (22.8)	
**Blood pressure control**						
Controlled	334 (66.8)	209 (71.8)	83 (68.6)	321 (73.1)	350 (78.8)	0.001
Out of control	166 (33.2)	82 (28.2)	38 (31.4)	118 (26.9)	94 (21.2)	
Means of systolic blood pressure levels	130 (18.6)	128 (15.4)	131 (15.1)	128 (16.5)	125 (17.7)	0.001^§^
Mean diastolic blood pressure levels	82 (11.3)	80 (9.14)	79 (10.0)	80 (9.9)	77 (10.8)	0.001^§^
Time of use of antihypertensive drugs (years)	4.5 (4.5)	3.6 (4.5)	4.8 (4.5)	2.6 (2.6)	4.7 (4.9)	0.001

*Differences in total N for each variable are due to missing values.^†^The Longitudinal Study of Adult Health (ELSA-Brasil).^1^Excessive drinking defined as >210 g alcohol/week for men and 140 g alcohol/week for women.^2^Reference values for blood pressure control: Controlled (<140/90 mmHg), Out of control (≥140 / 90 mmHg)^‡^p-value resulting from the Chi-square test^§^p-value resulting from the ANOVA test. Blood pressure (BP), Angiotensin converting enzyme inhibitors (ACEI), Angiotensin receptor blocker (ARB), Calcium channel blocker (BCC), Beta blocker (BB), Thiazide diuretic (Thiazide DIU)*

When investigating the association between self-reported race/skin color and BP control among users of ACEIs in monotherapy, even after adjusting for all variables, the chances of the Brown and Black populations having uncontrolled BP were 2.7 (95%CI: 1.7;4.3) and 2.2 (95%CI:1.3;3.4) higher, respectively, when compared to Whites. Among the users of ARBs, BBs, and thiazide DIUs, only Black individuals had a statistically higher chance of having uncontrolled BP when compared to Whites, after adjustment for confounding variables. Among CCB users, the self-reported race/skin color was not statistically associated with uncontrolled BP ( Table 3 ).

**Table 3:** – Crude and adjusted odds ratios (OR) * in blood pressure control+ of users of antihypertensive drugs in monotherapy in the baseline of the ELSA-Brasil1 2008-2010 (n=1.795)

Class of antihypertensive drugs	Multivariate			

	Model 0	Model 1	Model 2	Model 3
	**OR (95% CI)**	**OR (95% CI)**	**OR (95% CI)**	**OR (95% CI)**
**ACEI (n=500)**				
White	Ref.	Ref.	Ref.	Ref.
Brown	2.9^**^(1.9;4.5)	2.8^**^(1.8;4.4)	2.8^**^(1.8;4.4)	2.7^**^(1.7;4.3)
Black	2.5^**^(1.5;4.1)	2.3^**^(1.3;3.9)	2.3^**^(1.3;3.9)	2.2^**^(1.3;3.4)
**ARB (n=439)**				
White	Ref.	Ref.	Ref.	Ref.
Brown	1.3 (0.8;2.1)	1.1 (0.6;1.9)	1.1 (0.6;1.9)	1.2 (0.7;2.2)
Black	2.4^**^(1.25;4.51)	1.9 (0.9;4.0)	2.0 (1.0;4.1)	2.2^**^(1.0;4.7)
**BCC (n=121)**				
White	Ref.	Ref.	Ref.	Ref.
Brown	0.8 (0.3;2.1)	0.7 (0.3;1.9)	0.7 (0.2;1.9)	0.7 (0.2;2.1)
Black	1.3 (0.5;3.2)	1.0 (0.4;2.9)	1.0 (0.4;2.9)	1.1 (0.4;3.5)
**ΒB (n=444)**				
White	Ref.	Ref.	Ref.	Ref.
Brown	1.3 (0.8;2.3)	1.3 (0.7;2.3)	1.3 (0.7;2.3)	1.2 (0.6;2.2)
Black	2.3^**^(1.2;4.3)	2.1^**^(1.0;4.1)	2.1^**^(1.0;4.2)	2.1^**^(1.0:4.4)
**Thiazide DIU (n=291)**				
White	Ref.	Ref.	Ref.	Ref.
Brown	1.6 (0.9;3.0)	1.5 (0.8;2.9)	1.6 (0.8;3.2)	1.7 (0.9;3.4)
Black	2.2^**^(1.2:4.2)	1.9 (1.0;4.0)	2.1^**^(1.0;4.5)	2.4^**^(1.1;5.1)

* Odds Ratios (OR). + Reference category is blood pressure controlled (<140/90 mmHg)** p < 0,05. 1 The Longitudinal Study of Adult Health (ELSA-Brasil). Model 1: Adjusted for age, gender, and education. Model 2: Model 1 was then adjusted for excessive alcohol consumption. Model 3: Model 2 was adjusted for BMI, diabetes mellitus, and time of use of antihypertensive drugs. Angiotensin-converting enzyme inhibitors (ACEI), Angiotensin receptor blocker (ARB), Calcium channel blocker (BCC), Beta blocker (BB), Thiazide diuretic (Thiazide DIU)

## Discussion

This study innovates by investigating racial disparities in blood pressure control in monotherapy users of different classes of antihypertensive drugs in a sample with great racial diversity among adult Brazilian public servants. Our results do not corroborate with most of those found by the studies developed mainly with American populations,^8 , 12 , 13 , 26^ which show that Black users of ACEIs and ARBs have worse blood pressure control when compared to users of BBs, CCBs, and thiazide DIUs. Black users of antihypertensive drugs in monotherapy from the baseline of ELSA-Brasil, had a greater chance of having uncontrolled BP not only in the ACEI and ARB classes, but also in all others, with the exception of the CCB class.

The most recent American guideline for the treatment of hypertension^12^ recommends including a thiazide diuretics or calcium channel blockers for Black adults with hypertension without heart failure or chronic kidney disease. This recommendation is supported by results of studies carried out with an American population that have frequently shown that Black individuals, possibly because they have low renin production, have worse blood pressure control when treated with medications that act on the renin-angiotensin system. Furthermore, this population has worse cardiovascular outcomes when treated with these antihypertensive drugs.^3 , 4 , 27 , 28^

In addition to the lower production of renin among Black individuals, the lower response of ACEIs, compared to thiazide DIUs, CCBs, and BBs can be explained by other factors. It has been suggested that this lower response is attributed to a high sodium intake in Black individuals who are more sensitive to salt, in which the response to ACEIs would be somewhat weakened. Others have suggested that hypertension in the Black population may not have a mechanism independent of angiotensin.^29^ Moreover, this increase in sensitivity to salt may also explain the better control of blood pressure among Black users of thiazide DIUs.^30^ Other studies have shown a significant increase in the risk of adverse effects associated with ACEIs in Black individuals, for example coughing, which contributes to the greater discontinuation of treatment with ACEIs among this group when compared to other races.^31^

In the 1990s, a study by Saunders and collaborators showed that, among the Black population, the CCB class, as compared to BBs and ACEIs, was more effective in controlling both systolic and diastolic blood pressure levels.^32^ In addition, several other more recent studies mainly developed among Black Americans, have shown that CCBs, as compared to ACEIs, ARBs and BBs, were more effective in reducing the risk of several cardiovascular events, such as acute myocardial infarction, strokes, and cardiac insufficiency.^8 , 11 , 12 , 26^ In our study, among CCB users on monotherapy, Brown and Black individuals were not more likely to have uncontrolled blood pressure levels when compared to Whites. Although this lack of association can be explained by the low sampling power in this group (we have only 121 CCB users on monotherapy), the results are in line with the literature, which recommends CCBs as one of the first choices for the treatment of hypertension in the Black population.

In this sense, our results, which in summary showed that the greater chance of having uncontrolled BP in Black individuals is not restricted to users on ACEIs and ARBs in monotherapy, which is also found among users of thiazide DIUs and BBs, corroborate other studies that show that the possible explanations for Black individuals having worse BP control go beyond the physiological issue that would involve classes of medication. Socioeconomic differences, such as a low level of education, is one of the main determinants of the occurrence and the worse control of arterial hypertension,^33 , 34^ and may partly explain the differences between the Black, Brown, and White populations. In addition, social contexts or “neighborhoods” in which people live can contribute substantially to racial disparities in health^35 , 36^ and can play an important role in explaining the relationship between race/skin color and control of arterial hypertension.

In fact, previous studies developed at the ELSA-Brasil baseline have already shown racial disparities in the prevalence and control of hypertension. Chor et al. showed that individuals who claimed to be Black had poorer blood pressure control compared to those who claimed to be White, even among users of antihypertensive drugs.^16^ Barber et al. investigated the association between residential segregation and cardiometabolic risk factors, which included the presence of hypertension. The authors concluded that, despite having no statistically significant difference, the Black and Brown populations were more likely to live in economically segregated neighborhoods in relation to Whites and individuals who lived in these neighborhoods were 26% more likely to have hypertension.^36^ In addition, Baldo et al. also show that Black and Brown participants in the ELSA-Brasil baseline had greater arterial stiffness when compared to Whites. However, this difference was explained by the average blood pressure levels and the age of the participants, suggesting that therapeutic approaches should focus on the control of blood pressure levels, especially among Black individuals.^37^

It is important to highlight that, in our study, Black participants have the highest frequency of ACEI use, which would not be expected, since we tend to follow the guidelines based on Black American studies. However, the guidelines also recommend ACEIs or ARBs for individuals with diabetes,^11 , 12 , 22^ which can explain this result, since our Black participants have the highest frequency of DM.

Pena and colleagues showed that, in Brazil, skin color assessed phenotypically has a very weak correlation with the degree of ancestry.^38^ In this sense, ancestral results would help to better understand the racial disparities in the control of blood pressure from a genetic perspective. However, self-reported race/skin color is a phenotype that reaches beyond the genetics and the lived experience, thus reflecting the subjects’ perceptions of their own ethnic racial belonging.^39^

The present work innovates when investigating racial disparities in blood pressure control among users of different classes of antihypertensive drugs in a sample of adult Brazilian public servants; however it does have some limitations that should be highlighted. First, we had no information on the dose of antihypertensive treatment, and it is well-known that there are differences in dose optimization between different classes of the drug. Second, although monotherapy is more often used for milder cases, the staging of arterial hypertension can influence therapeutic options, with some classes more indicated at the beginning of treatment and others preferably in more advanced stages.^12^ However, there was no information on the hypertension staging. Third, although the uncontrolled BP was defined based on the values adopted by the national and international guidelines for the treatment and control of hypertension, it was based on a specific measurement of blood pressure levels. In this sense, false-positive and false-negative results can appear, which could interfere in our results.

Fourth, although the results are true for monotherapy, studies will show that the low effectiveness of ACEIs among Black individuals is reversed by the association of these medications with thiazide DIUs and CCBs.^40 , 41^ However due to the low sampling power, especially among new users, we have not tested combined therapy in the present study. Finally, although we have made adjustments for the main variables, this does not control unmeasured confounders.

## Conclusion

As far as we know, this is the first study to investigate racial disparities among users of different classes of antihypertensive drugs in monotherapy in a sample of Brazilian adults. In conclusion, our results suggest that the differences in blood pressure control between different racial groups are not explained by the possible lower effectiveness of ACEIs and ARBs in Black individuals, because this occurs within other classes of antihypertensive drugs. These results suggest caution in making antihypertensive treatment decisions based strictly on the race of the patients and provide relevant information that can guide decision-making for the treatment and control of arterial hypertension in the Brazilian context, suggesting that higher lack of BP control in Black individuals may be more related to social determinants than to the antihypertensive class used. Policies that act on adequate access to treatment and patient education should therefore be addressed.
